# Nanoparticle classification, physicochemical properties, characterization, and applications: a comprehensive review for biologists

**DOI:** 10.1186/s12951-022-01477-8

**Published:** 2022-06-07

**Authors:** Nadeem Joudeh, Dirk Linke

**Affiliations:** Department of Biosciences, University of Oslo, Blindern, P.O. Box 1066, 0316 Oslo, Norway

**Keywords:** Nanomaterials, Metal nanoparticles, Biogenic nanoparticles, Bionanoparticles, Nanobiotechnology, Characterization of nanomaterials

## Abstract

Interest in nanomaterials and especially nanoparticles has exploded in the past decades primarily due to their novel or enhanced physical and chemical properties compared to bulk material. These extraordinary properties have created a multitude of innovative applications in the fields of medicine and pharma, electronics, agriculture, chemical catalysis, food industry, and many others. More recently, nanoparticles are also being synthesized ‘biologically’ through the use of plant- or microorganism-mediated processes, as an environmentally friendly alternative to the expensive, energy-intensive, and potentially toxic physical and chemical synthesis methods. This transdisciplinary approach to nanoparticle synthesis requires that biologists and biotechnologists understand and learn to use the complex methodology needed to properly characterize these processes. This review targets a bio-oriented audience and summarizes the physico–chemical properties of nanoparticles, and methods used for their characterization. It highlights why nanomaterials are different compared to micro- or bulk materials. We try to provide a comprehensive overview of the different classes of nanoparticles and their novel or enhanced physicochemical properties including mechanical, thermal, magnetic, electronic, optical, and catalytic properties. A comprehensive list of the common methods and techniques used for the characterization and analysis of these properties is presented together with a large list of examples for biogenic nanoparticles that have been previously synthesized and characterized, including their application in the fields of medicine, electronics, agriculture, and food production. We hope that this makes the many different methods more accessible to the readers, and to help with identifying the proper methodology for any given nanoscience problem.

## Background

### Nano etymology

The prefix nano is derived from the Greek word nanos, “a dwarf”. In 1947, at the 14th conference of the International Union of Pure and Applied Chemistry (IUPAC), the prefix nano was officially adopted to describe the one-billionth part (10^–9^) of a unit^1^. In scientific literature, the prefix nano has been adopted as a popular label in many fields of modern science to describe small entities and processes. These terms include, but are not limited to nanoscience, nanotechnology, nanorobots, nanomagnets, nanoelectronics, nanoencapsulation, etc. [1]. In all of these cases, the prefix nano is used to describe “very small” entities or processes, most often at actual nanometer scale.

### Definitions

Nanoscience is a branch of science that comprises the study of properties of matter at the nanoscale, and particularly focuses on the unique, size-dependent properties of solid-state materials [2]. Nanotechnology is the branch that comprises the synthesis, engineering, and utilization of materials whose size ranges from 1 to 100 nm, known as nanomaterials [3]. The birth of nanoscience and nanotechnology concepts is usually linked to the famous lecture of Nobel laureate Richard Feynman at the 1959 meeting of the American Physical Society, ‘‘There’s Plenty of Room at the Bottom’’ [4]. However, the use of nanotechnology and nanomaterials goes back in history long before that.

### History of nanotechnology

Long before the era of nanotechnology, people were unknowingly coming across various nanosized objects and using nano-level processes. In ancient Egypt, dyeing hair in black was common and was for a long time believed to be based on plant products such as henna [5]. However, recent research on hair samples from ancient Egyptian burial sites showed that hair was dyed with paste from lime, lead oxide, and water [6]. In this dyeing process, galenite (lead sulfide, PbS) nanoparticles are formed. The ancient Egyptians were able to make the dyeing paste react with sulfur (part of hair keratin) and produce small PbS nanoparticles which provided even and steady dyeing.

Probably the most famous example for the ancient use of nanotechnology is the Lycurgus Cup (fourth century CE). This ancient roman cup possesses unusual optical properties; it changes its color based on the location of the light source. In natural light, the cup is green, but when it is illuminated from within (with a candle), it becomes red. The recent analysis of this cup showed that it contains 50–100 nm Au and Ag nanoparticles [7], which are responsible for the unusual coloring of the cup through the effects of plasmon excitation of electrons [8]. The ancient use of nanotechnology does not stop here, in fact, there is evidence for the early use of nanotechnology processes in Mesopotamia, Ancient India, and the Maya [9, 10].

### Why nanomaterials are different

Today, due to their unique properties, nanomaterials are used in a wide range of applications, such as catalysis, water treatment, energy storage, medicine, agriculture, etc*.* [11–13]. Two main factors cause nanomaterials to behave significantly differently than the same materials at larger dimensions: surface effects and quantum effects [14]. These factors make nanomaterials exhibit enhanced or novel mechanical, thermal, magnetic, electronic, optical, and catalytic properties [1, 15, 16].

Nanomaterials have different surface effects compared to micromaterials or bulk materials, mainly due to three reasons; (a) dispersed nanomaterials have a very large surface area and high particle number per mass unit, (b) the fraction of atoms at the surface in nanomaterials is increased, and (c) the atoms situated at the surface in nanomaterials have fewer direct neighbors [1, 14]. As a consequence of each of these differences, the chemical and physical properties of nanomaterials change compared to their larger-dimension counterparts. For instance, having fewer direct neighbor atoms for the atoms situated at the surface results in lowering the binding energy per atom for nanomaterials. This change directly affects the melting temperature of nanomaterials following the Gibbs–Thomson equation, e.g., the melting point of 2.5 nm gold nanoparticles is 407 degrees lower than the melting point of bulk gold [14]. Larger surface areas and larger surface-to-volume ratios generally increases the reactivity of nanomaterials due to the larger reaction surface [1], as well as resulting in significant effects of surface properties on their structure [17]. The dispersity of nanomaterials is a key factor for the surface effects. The strong attractive interactions between particles can result in the agglomeration and aggregation of nanomaterials, which negatively affects their surface area and their nanoscale properties [18]. Agglomeration can be prevented by increasing the zeta potential of nanomaterials (increasing the repulsive force) [19], optimizing the degree of hydrophilicity/hydrophobicity of the nanomaterial, or by optimizing the pH and the ionic strength of the suspension medium [20].

Nanomaterials display distinct size-dependent properties in the 1–100 nm range where quantum phenomena are involved. When the material radius approaches the asymptotic exciton Bohr radius (the separation distance between the electron and hole), the influence of quantum confinement becomes apparent [17]. In other words, by shrinking the size of the material, quantum effects become more pronounced, and nanomaterials become quantal. Those quantum structures are physical structures where all the charge carriers (electrons and holes) are confined within the physical dimensions [21]. As a result of quantum confinement effects, for instance, some non-magnetic materials in bulk such as palladium, platinum, and gold become magnetic in the nanoscale [14]. Quantum confinement can also result in significant changes in electron affinity or the ability to accept or donate electrical charges, which is directly reflected on the catalytic properties of the material. For example, the catalytic activity of cationic platinum clusters in N_2_O decomposition is dictated by the number of atoms in the cluster. 6–9, 11, 12, 15, and 20 atom-containing clusters are very reactive, while clusters with 10, 13, 14, and 19 atoms have low reactivity [14].

### Classification of nanomaterials

**Fig. 1:**
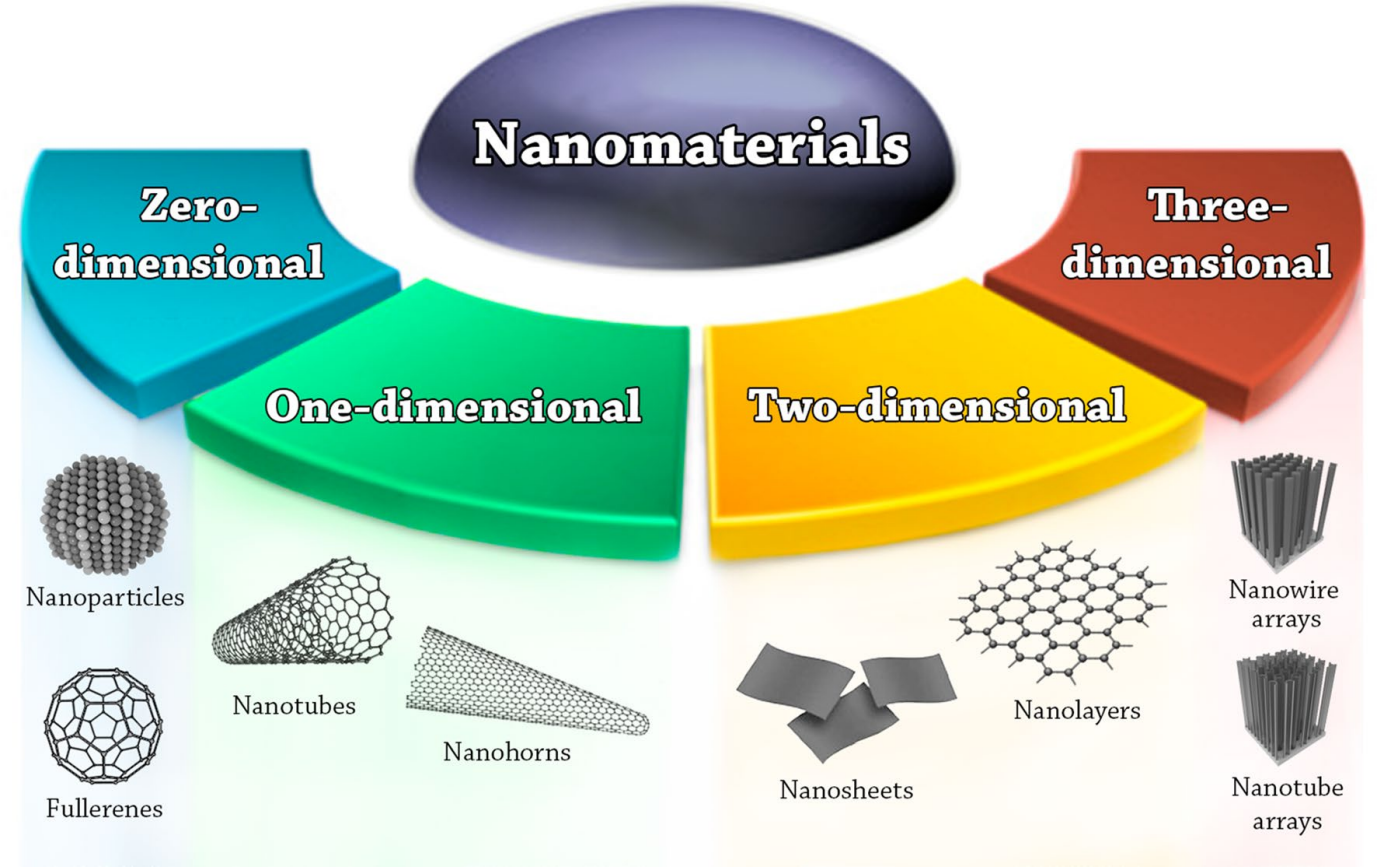
Nanomaterials classification based on dimensionality

The key elements of nanotechnology are the nanomaterials. Nanomaterials are defined as materials where at least one of their dimensions is in the nanoscale, i.e. smaller than 100 nm [22]. Based on their dimensionalities, nanomaterials are placed into four different classes, summarized in Fig. 1.

1. Zero-dimensional nanomaterials (0-D): the nanomaterials in this class have all their three dimensions in the nanoscale range. Examples are quantum dots, fullerenes, and nanoparticles.
2. One-dimensional nanomaterials (1-D): the nanomaterials in this class have one dimension outside the nanoscale. Examples are nanotubes, nanofibers, nanorods, nanowires, and nanohorns.
3. Two-dimensional nanomaterials (2-D): the nanomaterials in this class have two dimensions outside the nanoscale. Examples are nanosheets, nanofilms, and nanolayers.
4. Three-dimensional nanomaterials (3-D) or bulk nanomaterials: in this class the materials are not confined to the nanoscale in any dimension. This class contains bulk powders, dispersions of nanoparticles, arrays of nanowires and nanotubes, etc*.*

## Nanoparticles (NPs)

The International Organization for Standardization (ISO) defines nanoparticles as nano-objects with all external dimensions in the nanoscale, where the lengths of the longest and the shortest axes of the nano-object do not differ significantly. If the dimensions differ significantly (typically by more than three times), terms such as nanofibers or nanoplates maybe preferred to the term NPs^2^.

NPs can be of different shapes, sizes, and structures. They can be spherical, cylindrical, conical, tubular, hollow core, spiral, etc., or irregular [23]. The size of NPs can be anywhere from 1 to 100 nm. If the size of NPs gets lower than 1 nm, the term atom clusters is usually preferred. NPs can be crystalline with single or multi-crystal solids, or amorphous. NPs can be either loose or agglomerated [24].

NPs can be uniform, or can be composed of several layers. In the latter case, the layers often are: (a) The surface layer, which usually consists of a variety of small molecules, metal ions, surfactants, or polymers. (b) The shell layer, which is made of a chemically different material from the core layer. (c) The core layer, which is the central portion of the NP [25].

### Classification of NPs

Based on their composition, NPs are generally placed into three classes: organic, carbon-based, and inorganic [23].

#### Organic NPs

**Fig. 2:**
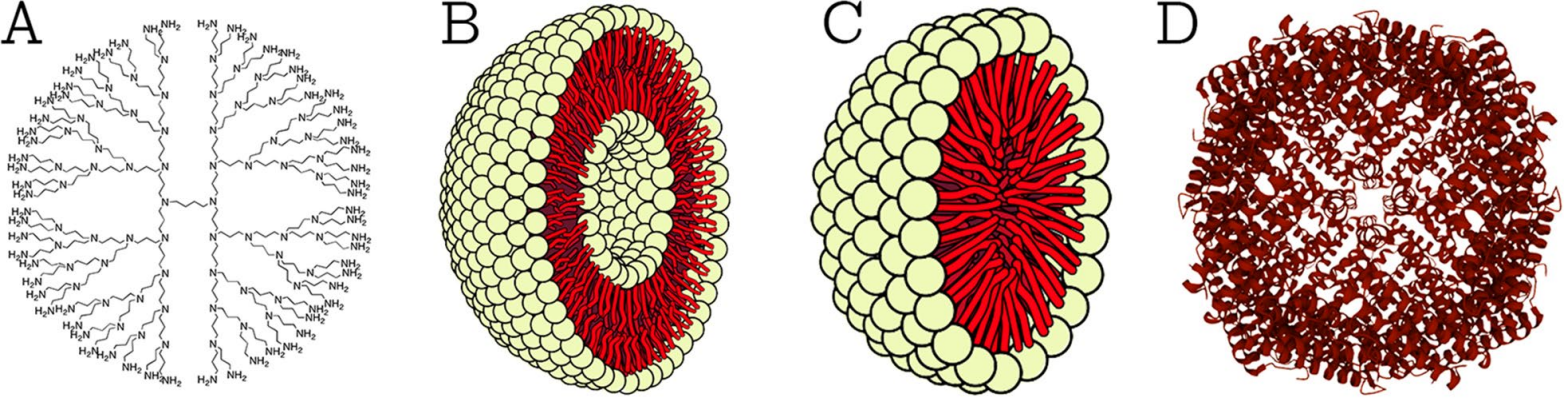
Types of organic NPs. **A** Dendrimers; **B** liposomes; **C** micelles; and **D** ferritin

This class comprises NPs that are made of proteins, carbohydrates, lipids, polymers, or any other organic compounds [26]. The most prominent examples of this class are dendrimers, liposomes, micelles, and protein complexes such as ferritin (shown in Fig. 2). These NPs are typically non-toxic, bio-degradable, and can in some cases, e.g., for liposomes, have a hollow core. Organic NPs are sensitive to thermal and electromagnetic radiation such as heat and light [23]. In addition, they are often formed by non-covalent intermolecular interactions, which makes them more labile in nature and offers a route for clearance from the body [27]. There are different parameters that determine the potential field of application of organic NPs, e.g., composition, surface morphology, stability, carrying capacity, etc*.* Today, organic NPs are mostly used in the biomedical field in targeted drug delivery [23] and cancer therapy [28].

#### Carbon-based NPs

**Fig. 3:**
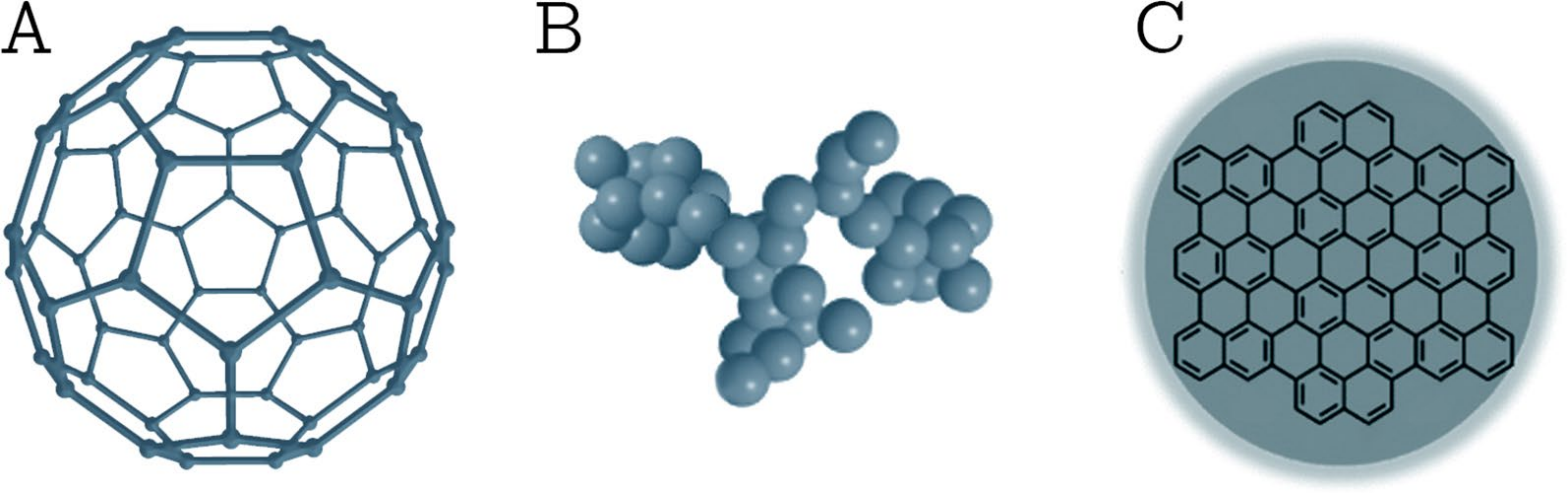
Different types of carbon-based NPs. **A** C_60_ fullerene; **B** carbon black NPs; and **C** carbon quantum dots

This class comprises NPs that are made solely from carbon atoms [23]. Famous examples of this class are fullerenes, carbon black NPs, and carbon quantum dots (shown in Fig. 3). Fullerenes are carbon molecules that are characterized by a symmetrical closed-cage structure. C_60_ fullerenes consist of 60 carbon atoms arranged in the shape of a soccer ball [29], but also other types of fullerenes such as C_70_ and C_540_ fullerenes have been described [30]. Carbon black NPs are grape-like aggregates of highly fused spherical particles [31]. Carbon quantum dots consist of discrete, quasi-spherical carbon NPs with sizes below 10 nm [32]. Carbon-based NPs unite the distinctive properties of sp^2^-hybridized carbon bonds with the unusual physicochemical properties at the nanoscale. Due to their unique electrical conductivity, high strength, electron affinity, optical, thermal, and sorption properties [25, 33], carbon-based NPs are used in a wide range of application such as drug delivery [34], energy storage [35], bioimaging [36], photovoltaic devices, and environmental sensing applications to monitor microbial ecology or to detect microbial pathogens [33]. Nanodiamonds and carbon nano onions are more complex, carbon-based NPs. Due to their characteristic low toxicity and biocompatibility, they are used in drug delivery and tissue engineering applications [37, 38].

#### Inorganic NPs

This class comprises NPs that not made of carbon or organic materials. The typical examples of this class are metal, ceramic, and semiconductor NPs. Metal NPs are purely made of metal precursors, they can be monometallic, bimetallic [39], or polymetallic [40]. Bimetallic NPs can be made from alloys or formed in different layers (core–shell) [39]. Due to the localized surface plasmon resonance characteristics, these NPs possess unique optical and electricals properties [25]. In addition, some metal NPs also possess unique thermal, magnetic, and biological properties [23]. This makes them increasingly important materials for the development of nanodevices that can be used in numerous physical, chemical, biological, biomedical, and pharmaceutical applications [41, 42] (these applications are discussed in detail later in the applications section of the review). In present days, the size-, shape-, and facet-controlled synthesis of metal NPs is important for creating cutting-edge materials [43].

Semiconductor NPs are made of semiconductor materials, which possess properties between metals and non-metals. These NPs possess unique wide bandgaps and show significant alteration in their properties with bandgap tuning compared to bulk semiconductor materials [25]. As a result, these NPs are important materials in photocatalysis, optic, and electronic devices [44, 45]. Ceramic NPs are inorganic solids made of carbonates, carbides, phosphates, and oxides of metals and metalloids, such as titanium and calcium [46]. They are usually synthesized via heat and successive cooling and they can be found in amorphous, polycrystalline, dense, porous or hollow forms [25]. They are mainly used in biomedical applications due to their high stability and high load capacity [47]. Nevertheless, they are also used in other applications such as catalysis, degradation of dyes, photonics and optoelectronics [46, 48].

### Physicochemical properties of NPs

As mentioned earlier, NPs can be used in a long list of applications due to their unique physical and chemical properties that do not exist in their larger-dimension counterparts of the same materials. The following sections summarize the most import physicochemical properties that are changing on the nanoscale.

#### Mechanical properties

Mechanical properties refer to the mechanical characteristics of a material under different conditions, environments, and various external forces. As for traditional materials, the mechanical properties of nanomaterials generally consist of ten parts: strength, brittleness, hardness, toughness, fatigue strength, plasticity, elasticity, ductility, rigidity, and yield stress [49]. Most inorganic, non-metallic materials are brittle materials and do not have significant toughness, plasticity, elasticity, or ductility properties. Organic materials on the other hand, are flexible materials and do not necessarily have brittleness and rigidity properties.

Due to surface and quantum effects, NPs display different mechanical properties compared to bulk materials [49]. For example, conventional FeAl powder which is composed of microparticles (larger than 4 µm), is brittle, while ultrafine FeAl alloy powder displays a good combination of strength and ductility as well as enhanced plasticity [50]. These new properties are believed to arise due to the diverse interaction forces between NPs or between them and a surface. The most important interaction forces involved are van der Waals forces, which consist of three parts, Keesom force, Debye force, and London force [51–53]. Other relevant interaction forces are electrostatic and electrical double layer forces, normal and lateral capillary forces, solvation, structural, and hydration forces [54].

There are different theories on how the interaction forces between NPs give them new mechanical properties, such as the DLVO (Derjaguin–Landau–Verwey–Overbeek) theory, JKR (Johnson–Kendall–Roberts) theory, and DMT (Derjaguin–Muller–Toporov) theory. The DLVO theory combines the effects of van der Waals attraction and electrostatic repulsion to describe the stability of colloidal dispersions [54]. This theory can explain many phenomena in colloidal science, such as the adsorption and the aggregation of NPs in aqueous solutions and the force between charged surfaces interacting through a liquid medium [55, 56]. Nevertheless, the DLVO theory is inadequate for the colloidal properties in the aggregated state [54].

When the size of objects decreases to the nanoscale, the surface forces become a major player in their adhesion, contact, and deformation behaviors. The JRK theory is applicable to easily deformable, large bodies with high surface energies, where it describes the domination of surface interactions by strong, short-range adhesion forces. In contrast to this, the DMT theory is applicable to very small and hard bodies with low surface energies, where it describes the adhesion being caused by the presence of weak, long-range attractive forces. Although the DLVO, JKR and DMT theories have been widely used to describe and study the mechanical properties of NPs [57, 58], it is still a matter of debate whether or not continuum mechanics can be used to describe a particle or collection of particles at the nanometer scale [54].

#### Thermal properties

Heat transfer in NPs primarily depends on energy conduction due to electrons as well as photons (lattice vibration) and the scattering effects that accompany both [59]. The major components of thermal properties of a material are thermal conductivity, thermoelectric power, heat capacity, and thermal stability [59, 60].

NP size has a direct impact on electrical and thermal conductivity of NPs [60]. As the NP size decreases, the ratio of particle surface area respective to its volume increases hyperbolically [60]. Since the conduction of electrons is one of the two main ways in which heat is transferred, the higher surface-to-volume ratio in NPs provides higher number of electrons for heat transfer compared to bulk materials [61]. Moreover, thermal conductivity in NPs is also promoted by microconvection, which results from the Brownian motion of NPs [62]. Nevertheless, this phenomenon only happens when solid NPs are dispersed in a liquid (generating a Nanofluid) [63]. As an example, the addition of Cu NPs to ethylene glycol enhances the thermal conductivity of the fluid up to 40% [64].

The thermoelectric power of a material depends on its Seebeck coefficient and electrical conductivity ($P={S}^{2}\sigma$, where P is thermoelectric power, S is the Seebeck coefficient, and $\sigma$ is the electrical conductivity). The scattering of NPs in bulk materials (doping) is known to enhance the thermoelectric power factor [65]. This enhancement could come from the enhancement of the Seebeck coefficient or the enhancement of electrical conductivity. The embedding of size-controlled NPs in bulk thermoelectric materials helps to reduce the lattice thermal conductivity and enhances the Seebeck coefficient due to electron energy filtering [66, 67]. Generally, the enhancement of electrical conductivity is accompanied by the reduction of the Seebeck coefficient and vice versa [65] However, the doping of InGaAlAs material with 2–3 nm Er NPs resulted in the significant increase of thermoelectric power of the material through the enhancement of the conductivity while keeping the Seebeck coefficient unchanged [65]. Depending on NP size, volume fraction, and band offset, a NP-doped sample can either enhance or suppress the electrical conductivity in comparison with undoped bulk sample.

Experimental studies have shown that the heat capacity of NPs exceeds the values of analogous bulk materials by up to 10% [68], e.g. in the case of Al_2_O_3_ and SiO_2_ NPs [69, 70]. The major contribution to heat capacity at ambient temperatures is determined by the vibration degrees of freedom, i.e., the peculiarities of phonon spectra (vibrational energy that arises from oscillating atoms within a crystal) are responsible for the anomalous behavior of heat capacity of NPs [68]. NPs usually exhibit a significant decrease in melting temperature compared to their analogous bulk materials [71]. The main reason for this phenomenon is that the liquid/vapor interface energy is generally lower than the average solid/vapor interface energy [72]. When the particle size decreases, its surface-to-volume ratio increases, and the melting temperature decreases as a result of the improved free energy at the particle surface [73]. For instance, the melting temperature of 3 nm Au NPs is 300 degrees lower than the melting temperature of bulk gold [14]. In addition, NP composition plays an important role in thermal stability. For example, the thermal stability of Au in Au_0.8_Fe_0.2_ is significantly higher than of pure Au or Au_0.2_Fe_0.8_ [74]. Generally, bimetallic alloy NPs show higher thermal stabilities and melting temperatures than monometallic NPs due to the alloying effect [75, 76].

#### Magnetic properties

All magnetic compounds include a ‘magnetic element’ in their formula, i.e., Fe, Co, or Ni (at ambient temperatures). There are only three known exceptions that are made from mixed diamagnetic elements, Sc_3_In, ZrZn_2_, and TiBe_2-x_Cu_x_ [77–80]. Otherwise, elements such as Pd, Au, or Ag are diamagnetic. This all changes in the nanoscale. Several materials become magnetic in the form of NPs as a result of uneven electronic distribution [25]. For instance, FeAl is not magnetic in bulk but in the form of NPs, it is becomes magnetic [50], other examples include Pd and Au [81]. In bulk materials, the key parameters for determining magnetic properties are composition, crystallographic structure, magnetic anisotropy, and vacancy defects [82, 83]. However, on the nanoscale, two more important parameters are strongly involved, i.e., size and shape [84].

One of the interesting size-dependent phenomena of NPs is superparamagnetism [84]. As the size of the NPs decreases, the magnetic anisotropy energy per NP decreases. The magnetic anisotropy energy is the energy keeping the magnetic moment in a particular orientation. At a characteristic size for each type of NPs, the anisotropy energy becomes equal to the thermal energy, which allows the random flipping of the magnetic moment [85], in this case, the NP is defined as being superparamagnetic [86]. Superparamagnetic NPs display high magnetization only in the presence of a magnetic field, and once it is removed they do not retain any magnetization [87]. Superparamagnetism was long believed to form only in small ferromagnetic or ferrimagnetic NPs [88], but interestingly, other paramagnetic materials show magnetism in the nanoscale too [81].

**Fig. 4:**
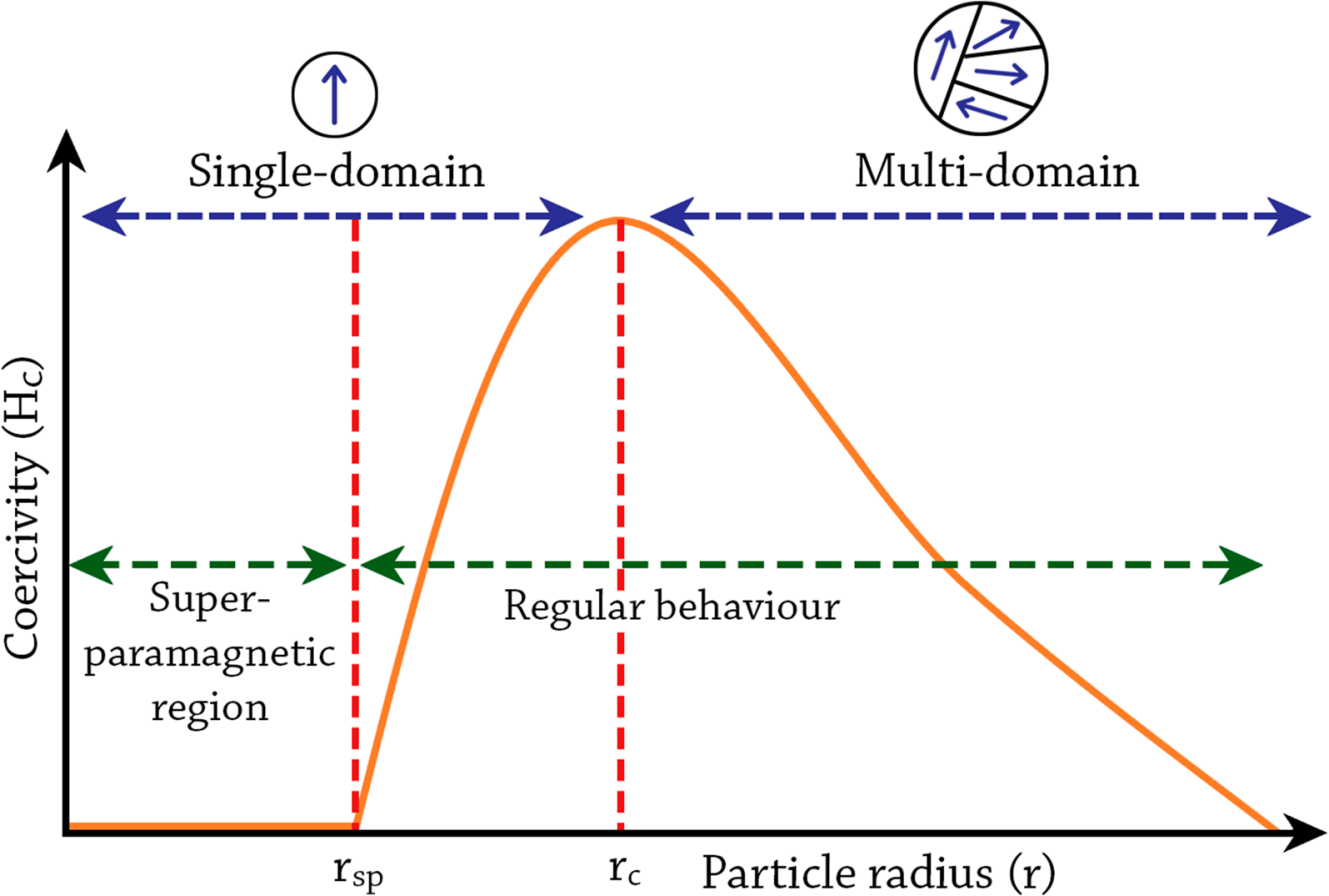
The change in magnetic coercivity of NPs as a function of particle radius. Figure adapted from Kalubowilage et al., 2019 [89]. *rc* critical radius, *rsp* threshold radius for superparamagnetism

NP size effects can also be observed in changes in magnetic coercivity, i.e., the resistance of a magnetic material to changes in magnetization (Fig. 4). In contrast to large particles or bulk materials, which possess multiple magnetic domain structures, small NPs possess single magnetic domain structures below a certain critical radius (r_c_), where all magnetic spins in the NP align unidirectionally (blue arrows in Fig. 4). However, the NP radius has to be lower than the threshold radius for superparamagnetism (r_sp_) in order to be superparamagnetic [89]. In the single-domain regime, between r_sp_ and r_c_, the magnetic coercivity increases as the size of the NP increases until it reaches the maximum at r_c_ [84]. In this size regime, due to the high magnetic coercivity, the NPs behave similarly as their larger dimension counterparts despite having a single domain structure, i.e., they become ferromagnetic for ferromagnetic materials or paramagnetic for paramagnetic materials etc*.* Above r_c_, the magnetic coercivity starts to decrease when multiple magnetic domains are formed in a single NP. The critical radius represents the size where it is energetically favored for the NP to exist without a domain wall [86]. The calculated critical radii for some common magnetic materials are 35 nm of Ni, 8 nm for Co, and 1 nm for Fe [90]. Above that point, multi-domain magnetism begins in which a smaller reversal magnetic field is required to make the net magnetization zero [84].

The second key parameter for determining the magnetic properties of NPs is the shape of NPs. In comparison to the size parameter, there is significant less research on the effect of shape on the magnetic properties of NPs having the same volume [86]. However, large differences in coercivity were found between a set of cubic and spherical CoFe_2_O_4_ NPs [91]. Unlike the curved topography in spherical CoFe_2_O_4_ NPs, cubic CoFe_2_O_4_ NPs have fewer missing oxygen atoms, and it was hypothesized that this led to less surface pinning and to lower coercivity for the cubic structures [86]. Other studies also found differences in magnetism between spherical and cubic Fe_3_O_4_ NPs [92, 93].

Similar to bulk materials, the composition also affects the magnetism of NPs. The magnetocrystalline phase of the NP is significant in determining its magnetic coercivity [94]. This effect can be observed in magnetic bimetallic core–shell or alloy NPs with anisotropic crystalline structures. For example, Co@Pt core–shell NPs composed of an isotropically structured face-centered cubic Co core and a non-magnetic Pt shell exhibit superparamagnetic behavior with zero coercivity at room temperature [95]. In general, the compositional modification of NPs by the adoption of magnetic dopants is known to significantly change the magnetism of NPs [96].

#### Electronic and optical properties

Metallic and semiconductor NPs possess interesting linear absorption, photoluminescence emission, and nonlinear optical properties due to the quantum confinement and localized surface plasmon resonance (LSPR) effect [97, 98]. LSPR phenomena arise when the incident photon frequency is constant with the collective excitation of the conductive electrons [25].Due to this phenomenon, noble metal NPs exhibit a strong size-dependent UV–visible extinction band that is not present in the spectra of bulk metals. Generally, the optical properties of NPs depend on the size, shape, and the dielectric environment of the NPs [99].

**Fig. 5:**
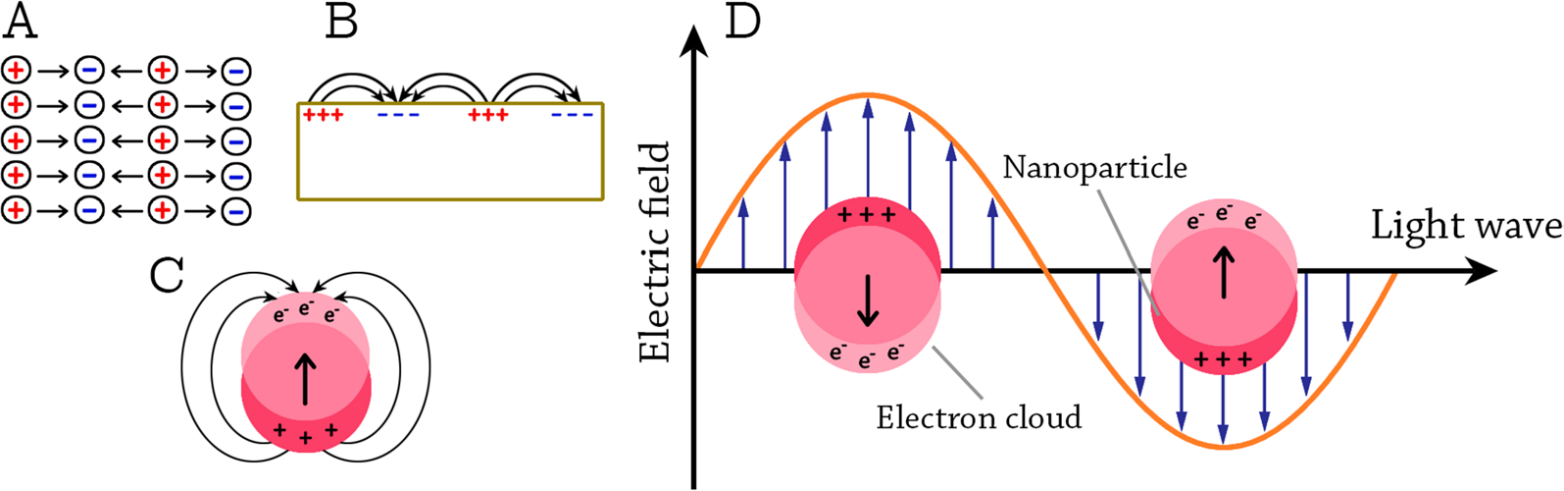
Graphical illustration of the types of plasmons. **A** bulk; **B** surface propagating; and **C** surface localized plasmons (adapted from Khlebtsov et al., 2010 [98]). **D** graphical illustration of the localized surface plasmon resonance (LSPR) in NPs (adapted from Kelly et al., 2003 [99])

The collective excitations of conductive electrons in metals are called plasmons [100]. Depending on the boundary conditions, bulk plasmons, surface-propagating plasmons, and surface-localized plasmons are distinguished (Fig. 5A–C). Because of their longitudinal nature, the bulk plasmons cannot be excited by visible light. The surface-propagating plasmons propagate along metal surfaces in a waveguide-like fashion [98]. In the case of NPs, when they are irradiated by visible light, the oscillating electric field causes the conductive electrons to oscillate coherently. When the electron cloud is displaced relative to the nuclei, a restoring force rises from Coulomb attraction between electrons and nuclei that results in oscillation of the electron cloud relative to the nuclear framework [99]. This creates uncompensated charges at the NP surface (Fig. 5D). As the main effect producing the restoring force is the polarization of the NP surface, these oscillations are called surface plasmons and have a well-defined resonance frequency [98].

Experimental studies on Ag NPs showed significant differences in their optical properties based on the size of NPs. For Ag NPs with 30 nm radius, the main extinction peak was at 369 nm wavelength, while for Ag NPs with 60 nm radius, a totally different behavior was observed [99]. The same researchers found that the shape of the NPs also is critical for the optical properties, the plasmon resonance wavelength shifts to the red as the NPs become more oblate [99], demonstrating that plasmon resonance strongly depend on NPs shape. With respect to the dielectric environment of the NPs, both the surrounding solvent and the support (substrate) were found to be critical for the optical properties. For Ag NPs, both experimental and theorical studies on the effect of surrounding solvent show that plasmon wavelength linearly depends on the refractive index of the solvent [99, 101]. At the same time, 10 nm Ag NPs supported on mica substrates displayed LSPR wavelength shifts to the red compared to unsupported NPs [102]. The biogenic synthesis of NPs can also improve the optical properties. Biologically produced CeO_2_ NPs using *Simarouba glauca* leave extract were found to have different absorption bands and higher band gap energies compared to chemically produced CeO_2_ NPs. These superior optical properties were attributed to the better crystallinity and small size of biogenic NPs compared to chemical NPs [103]. Biogenic NPs can also offer higher photocatalytic activities, e.g., ZnO NPs produced by *Plectranthus amboinicus* leaf extract had higher photocatalytic activity in the photodegradation of methyl red under UV illumination compared to chemical produced ZnO NPs [104].

#### Catalytic properties

Nano-catalysis, i.e., the use of NPs as catalysts, is a quickly evolving field within chemical catalysis. Significantly enhanced or novel catalytic properties such as reactivity and selectivity have been reported for NP catalysts compared to their bulk analogues. The catalytic properties of NPs depend on the size, shape, composition, interparticle spacing, the oxidation state, and the support of the NPs [76].

The dependency of catalytic activity on the size of NPs is well studied. The relation is an inverse one, i.e., the smaller the NPs the more catalytically active they are. This relationship was found e.g., in the electro-catalysis oxidation of CO by size-selected Au NPs (1.5, 4, and 6 nm) deposited on indium tin oxide. The researchers observed that the smallest NPs provided the highest normalized current densities [105]. The same relationship was also found in several other studies [106–110]. Goodman et al., 1998 [111] speculated originally that this behavior could be attributed to quantum-size effects generated by the confinement of electrons within a small volume. Later, size-dependent changes in the electronic structure of the clusters [112] and the resulting larger number of low-coordinated atoms available for interaction by the larger surface-to-volume ratios with smaller NPs were discussed [76].

The shape is also known to affect the reactivity and selectivity of the NPs. For the oxidation of CO by Au NPs, hemispherical NPs were found to be more active than spherical ones [113]. For the oxidation of styrene by Ag NPs, nanocubes were found to be fourteen times more efficient than nanoplates and four times more efficient than nanospheres [114]. The reason for these dramatical changes are attributed to the increase/decrease in the relative area of the catalytically active surface facets [76] or to the differences in stability for different NP shapes [115].

As for composition, several studies have shown that the use of alloys in NPs can enhance the catalytic activity as a result of the alloying effect causing changes in the electronic properties of the catalyst, decreasing poisoning effects, and providing distinct selectivities [76]. For example, the alloying of Pt with other metals such as Ru, Ni, and Co, was reported to enhance the hydrogenation and oxygen reduction activity of the NP catalyst material, as well as enhancing the resistance against CO poisoning [116–118]. However, the alloying of Pt with Fe, Ru, and Pd, resulted in reduced reactivity for methanol decomposition [119]. This reduction in reactivity was explained by the possible occupation of the surface with the addition metal atoms, since pure Fe, Ru, and Pd clusters are less reactive for methanol decomposition than similarly-sized pure Pt clusters. In general, the change in the composition of NPs changes the electronic structure of metal surfaces by the formation of bimetallic bonds as well as the modification of metal–metal bond lengths [76]. In addition, the charge-transfer phenomenon between different metals may favorably change the binding energy of adsorbents, lower the barriers for specific chemical reactions, and enhance resistance against poisoning [120–122].

The catalytic activity and stability of 2 nm Au NPs dispersed on polycrystalline TiC films displayed a strong dependence on interparticle spacing. In this study, Au NPs having two different interparticle spacing (30 and 80 nm) were analyzed by Thermal Desorption Spectroscopy. It was found that the sample with smaller interparticle spacing was poisoned and subsequently deactivated while the sample with longer interparticle spacing showed longer lifetime [123]. At the same time, the oxidation state of NPs was shown to affect the catalytic activities. Ru NPs under rich O_2_ conditions and moderate temperatures oxidize and form RuO_2_, the reaction of CO oxidation was found to occur on the metal oxide surface not the metal surface [124]. A similar effect on CO oxidation was also observed with Pt NPs in which the reactivity of PtO_2_ was found to be higher than Pt [125]. The reaction of CO oxidation was compared for several metal NPs (Ru, Pd, Ir, Os, and Pt) and their corresponding oxides, and the oxides were indeed more reactive than the metals [126, 127]. The superior catalytic performance of RuO_2_ over their metallic counterparts is generally agreed on, nevertheless, the same cannot be said for other catalytically active metals such as Pt [76]. In general, these differences in catalytic performance are attributed to the electron transfer processes at the metal/metal oxide interfaces. Consequently, the view that NP oxidation is an undesirable process that leads to the reduction of catalytic performance needs to be reconsidered [128].

An example for the effect of the support material is the role of the MgO support for Au NPs, where MgO was found to be important for CO oxidation and particularly, for controlling the rate of CO oxidation through oxygen vacancies [129]. Later, the process of electron charge transfer from oxygen vacancies at the metal-substrate interface of supported Au NPs was suggested to be an ideal environment for O_2_ activation and oxidation reactions [130]. A similar behavior was also found in the decomposition of SO_2_ and dissociation of water by Au NPs supported on CeO_2_, in which CeO_2_ supports played a critical role [131]. The experiments showed that not only the chemical composition of the support affects the reactivity of the catalyst, but the crystal structure of the support, too [132]. Enhanced catalytic performance for CO oxidation and SO_2_ dissociation have also been reported for Au NPs supported on metal carbides such as TiC [108, 133]. In addition to enhanced catalytic reactivities, the support also plays an important role in NP stabilization [106], i.e., the stabilization of NPs against coarsening, the stabilization of metal oxides at the NP surface, and the stabilization of intermediate reactions species [76].

### Characterization of NPs

The properties of NPs determine their potential applications. Hence, different methods and techniques are used for the analysis and characterization of the various physicochemical properties of NPs. Table 1 summarizes all characterization techniques mentioned in this review and shows what properties and features can be resolved by each technique.

**Table 1:** Common techniques and methods used for NP characterization

	Technique	Properties and features that can be resolved by each technique									
		Size	Shape	Dispersity	Localization	Agglomeration	Surface morphology		Surface area	Pore size	Literature
Morphological and topographical	TEM	✓	✓	✓	✓	✓					[137, 309]
	SEM	✓	✓	✓		✓					[138, 310]
	STM						✓				[139, 311]
	DLS	✓		✓							[142–144]
	NTA	✓		✓							[147, 148]
	BET								✓		[150–153]
	BJH									✓	[150–153]

	Technique	Composition	Phase	Crystallinity	Functionalization	Oxidation	Surface charge	Polarity	Bonding	Electrochemical	Literature
Structural and chemical	XRD		✓	✓							[142, 153]
	EDX	✓									[161–164]
	HAADF	✓									[167–169]
	XPS	✓				✓			✓	✓	[172–175]
	FTIR	✓			✓	✓		✓	✓		[179, 180]
	Zeta potential						✓				[182–186]
	CV					✓				✓	[190, 191]
	Raman spectroscopy			✓					✓		[195, 196]

	Technique	Toughness	Tensile strength	Compressive strength	Elasticity	Viscoelasticity	Hardness	Stiffness			Literature
Mechanical	UTM		✓	✓	✓						[255, 256]
	Nano-indentation	✓		✓	✓		✓				[258, 259]
	DMA					✓		✓			[261, 262]

	Technique	Absorption	Reflectance	Fluorescence	Luminescence	Bandgap	Electronic state	Photoactivity	Electrical conductance		Literature
Optical, electronic, and electrical	Raman spectroscopy						✓				[195, 196]
	SERS						✓				[197]
	UV–vis	✓	✓			✓					[200–204]
	PL			✓	✓						[200–204]
	DRS					✓	✓	✓	✓		[206–208]
	Ellipsometry	✓	✓						✓		[211, 212]

	Technique	Saturation magnetization		Remnant magnetization	Coercivity	g-factor	Magnetic field intensity	Magnetic force	Magnetic susceptibility		Literature
Magnetic	MFM					✓	✓	✓			[216–218]
	VSM	✓		✓	✓						[221, 222]
	SQUID	✓		✓	✓					✓`	[226, 227]
	ESR					✓					[231, 232]

	Technique	Melting point		Crystallization point	Structural-phase transition	Heat capacity		Thermal conductivity	Thermal Stability	Oxidative stability	Literature
Thermal	DSC	✓		✓	✓	✓				✓	[238, 239]
	DTA	✓		✓	✓					✓	[249, 250]
	TGA	✓			✓				✓		[249, 250]
	THW							✓	✓		[252, 253]

The physicochemical properties and features that can be resolved by each technique or method are shown together with examples of experimental research from literature

#### Morphological and topographical characterization

The morphological and topographical features of NPs are of great interest since they influence most of the properties of NPs as described above. These features include the size, shape, dispersity, localization, agglomeration/aggregation, surface morphology, surface area, and porosity of the NPs. The following techniques are regularly used for the characterization of morphological and topographical features of NPs.

##### Electron microscopy (EM)

Scanning electron microscopy (SEM), scanning tunneling microscopy (STM), and transmission electron microscopy (TEM) are frequently employed for the analysis of NP size, shape, and surface. In SEM, an electron gun is used to produce a beam of electrons that is controlled by a set of lenses to follows a vertical path through the microscope until it hits the samples. Once the sample is hit by the beam, electrons and X-rays are ejected from the sample. Detectors are then used to collect the X-rays and scattered electrons in order to create a 3D image of the sample. SEM provides different information about the NPs such as size, shape, aggregation, and dispersion [134]. Similarly, TEM provides information about the size, shape, localization, dispersity, and aggregation of NPs in two-dimensional images [25]. TEM employs an electromagnetic lens that focuses a very fine beam of electrons into an ultrathin section of the sample. This beam passes through the specimen where the electrons either scatter or penetrate the sample and hit a fluorescent screen at the bottom of the microscope. The difference in electron densities is used for the contrast to create an image of the specimen. TEM can be also used for the characterization of NP crystal structure through the use of selected area electron diffraction (SAED), where the electron beam is focused on a selected area in the sample and the scattered electrons are used to obtain a diffraction pattern. STM is based on the phenomenon of quantum tunneling, where a metallic tip is brough very close to the sample surface and used to apply voltage. When voltage is applied, electrons from the sample surface are extracted creating an electrical current that is used to reconstruct an image of the surface with atomic resolution [135]. STM is mainly used to characterize the topography of NPs. For inorganic NPs, these techniques offer excellent approaches for the determination of morphological features of NPs. For organic NPs (or NPs coated with biological materials), these techniques require sophisticated sample preparations which constitute major restrictions to their use [136]. The sample preparation for these techniques might cause sample dehydration, which might lead e.g. to sample shrinking and aggregation [136].

Examples: TEM was used for the characterization of Ag NPs produced by *Arbutus unedo* leaf extract. In this example, the NPs have a spherical morphology with a uniform size of 30 nm. The NPs were found to agglomerate into small aggregates, each including 5–6 NPs. At the same time, the SAED approach was used to determine the crystal structure of the NPs. The majority of the NPs were found to be single crystalline cubic materials predominately oriented along their (111) direction [137]. For the characterization of Ag NPs produced by *Diospyros kaki* leaf extract, SEM helped to show that the NPs were also spherical and the size was 32 nm with some deviations [138]. STM is less frequently used for the characterization of biogenic NPs. The features of Ag NPs produced by lime, sweet-lime, and orange juices were compared using STM technique [139].

##### Dynamic light scattering (DLS)

This technique is a common approach for the analysis of NP size and size distribution. This approach involves the measurement of light interference based on the Brownian motion of NPs in suspension, and on the correlation of NP velocity (diffusion coefficient) with their size using Strokes-Einstein equation [140]. The size distribution range of NPs is shown as the polydispersity index, which is the output of an autocorrelation function [136]. The polydispersity index values lie between 0 and 1, where 0 represents a completely homogenous population and 1 represents a highly heterogeneous population. This technique also allows the analysis of non-spherical NPs through the use of multistage DLS [136]. This technique is also referred to as photon correlation spectroscopy (PCS) [141].

Examples: DLS was used to measure the size and the size distribution profile of a wide range of biogenic NPs. The average size of Ag NPs produced by *Trichoderma koningii* fungi was found to be around 25 nm and the size distribution profile was between 14 and 34 nm. The polydispersity index for those NPs was 0.681, which indicates that they are polydispersed [142]. While the average size of Ag NPs produced by potato (*Solanum tuberosum*) was found to be around 10–12 nm with a wider distribution profile between 3–65 nm [143]. In a different application, DLS was employed to study the size increase of biogenic MnO_2_ NPs overtime, demonstrating that their size is 7.5 nm after 3 min of the initiation of the reaction, then their size grows overtime until it become 54 nm after 31 min [144].

##### Nanoparticle tracking analysis (NTA)

This method is used for the analysis of NP size in suspensions based on their Brownian motion. Like in DLS, the rate of NP movement is correlated with their size using Strokes-Einstein equation, allowing the measurement of size distribution profiles for NPs with 10–1000 nm diameter. Its advantage over DLS is that NP motion is analyzed by video. Individual positional changes of NPs are tracked in two dimensions, which are used to determine NP diffusion rates, and by knowing the diffusion coefficient, the hydrodynamic diameter of the particles can be calculated. In DLS, individual NPs are not visualized, but instead, the time-dependent intensity fluctuations caused by Brownian motion are used to calculate the polydispersity index [145]. NTA was found to be more precise for sizing monodisperse as well as polydisperse organic NPs compared to DLS [146].

Examples: NTA was used to measure the size and dispersity of Ag NPs produced by *Camellia sinensis* (green tea) powder, the NPs were found to be well dispersed in an aqueous medium with an average size of 45 ± 12 nm [147]. For Se NPs produced by lactic acid bacteria, NTA was employed to measure the size and the concentration of NPs. The average size was found to be 187 ± 56 nm with a concentration of (4.67 ± 0.30) × 10^9^ Se NPs per ml [148].

##### Brunauer–Emmett–Teller (BET) method

**Fig. 6:**
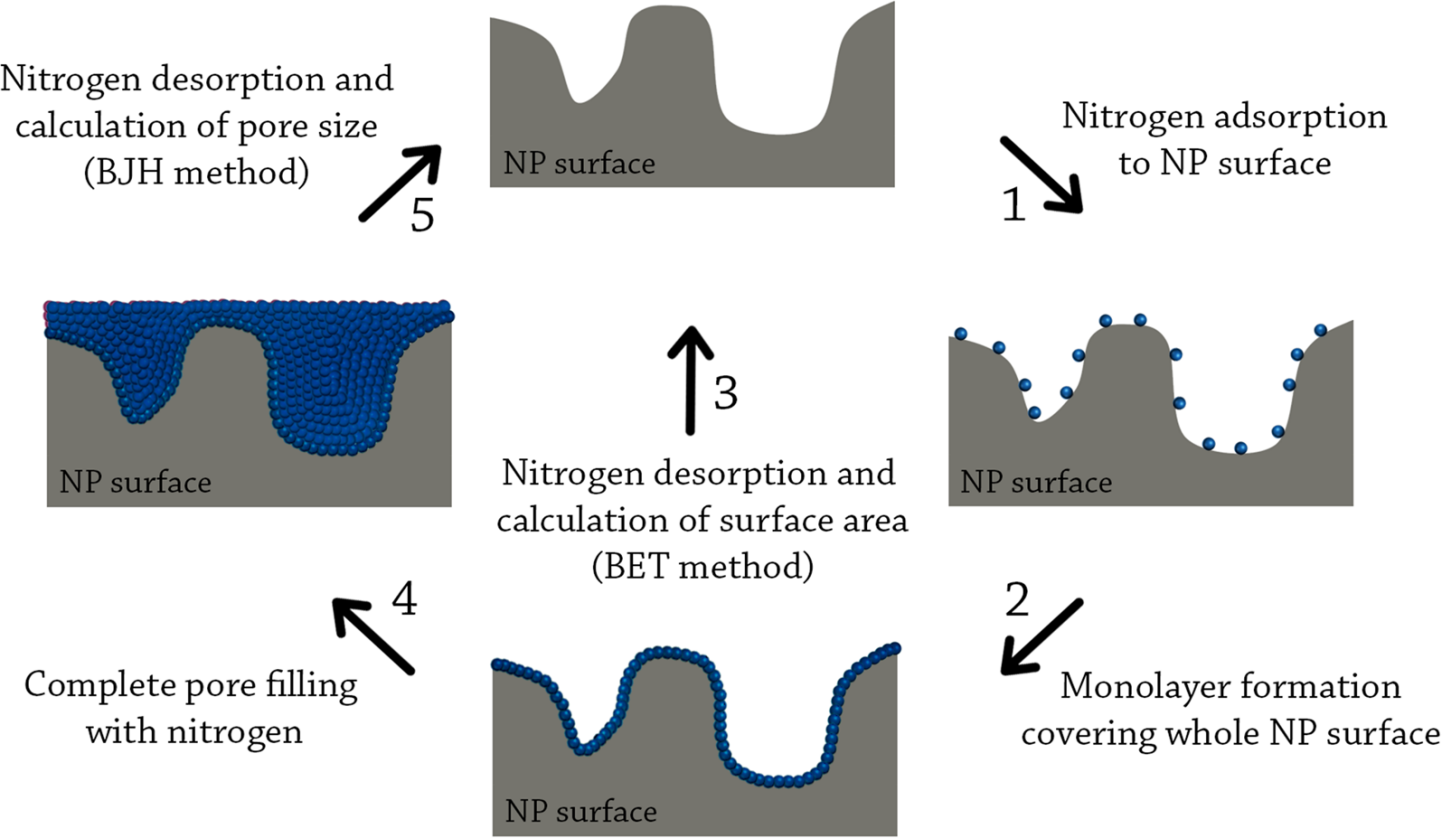
Principles of the BET and BJH methods. The BET method (steps 1–3) is based on the adsorption of nitrogen on the NP surface. After the formation of a monolayer, nitrogen is desorbed, and the surface area is calculated. The BJH method (steps 1, 2, 4, and 5) is based on the complete filling of NP pores with liquid nitrogen. When saturation is reached, nitrogen is desorbed, and pore size is calculated

This method is based on the adsorption and desorption principle developed by Stephen Brunauer, Paul Emmett, and Edward Teller, and it is considered one of the best methods for the analysis of NP surface area [25]. In BET analysis, a partial vacuum is created to produce adsorption between the sample and liquid N_2_ (because the interaction between solid and gaseous phases is weak, the surface is cooled with liquid N_2_ to obtain detectable amounts of adsorption). After the formation of adsorption monolayers, the sample is removed from the N_2_ atmosphere and heated to cause the adsorbed N_2_ to be released from the material (desorption) and quantified. The data collected is displayed in the form of isotherms (graphs representing the amount of N_2_ adsorbed as a function of relative pressure at a constant temperature). The data is displayed in five isotherms where the information is used to determine the surface area of the sample [25, 149]. Figure 6 graphically illustrates the principle of this method.

Examples: The BET method was employed to measure the surface area of CeO_2_ NPs produced by *Eucalyptus globulus* leaf extract. The surface area was found to be 40.96 m^2^/g of biogenic CeO_2_ NPs, much higher than the commercial CeO_2_ NPs (8.5 m^2^/g) [150]. BET was also used to measure the surface area of SiO_2_ NPs produced by rice husk, CuO NPs produced by *Leucaena leucocephala* leaf extract, and Ag NPs produced by *Acanthospermum hispidum* leaf extract. In these examples, the surface area was 7.15 m^2^/g, 47.54 m^2^/g, and 9.91 m^2^/g, respectively [151–153].

##### Barrett–Joyner–Halenda (BJH) method

This method is based on the Barrett–Joyner–Halenda principle and is used for the determination of porosity (or pore size) of NPs. Similar to the BET method, this method also involves the use of N_2_ gas to adsorb to the sample. In the BJH method, the process is extended so the gas condensates in the sample pores as pressure increases. The pressure is increased until a saturation point is achieved, at which all the pores of the sample are filled with liquid. Afterwards, the condensated gas is allowed to evaporate where the desorption data is calculated and correlated to the pore size using a modified Kelvin equation (Kelvin model of pore filling) [154, 155]. Figure 6 graphically illustrates this method.

Examples: The BJH method was employed to study the pore size of a wide range of biogenic NPs, for instance, the pore size of CeO_2_ NPs produced by *Eucalyptus globulus* leaf extract was found to be 7.8 nm [150], the pore size of CuO NPs produced by *Leucaena leucocephala* leaf extract was 2.13 nm [152], the pore size of SiO_2_ NPs produced by rice husk and Ag NPs produced by *Acanthospermum hispidum* leaf extract were much larger, being 29.63 nm and 36.34 nm, respectively [151, 153].

#### Structural and chemical characterization

The structural characterization of NPs and the study of their composition is of high interest due to the strong influence of these parameters on the physicochemical properties. The following techniques are commonly used for the analysis of NP composition, phase, crystallinity, functionalization, chemical state (oxidation), surface charge, polarity, bonding, and electrochemical properties.

##### X-ray diffraction analysis (XRD)

This technique is based on irradiating a material with incident X-rays and then measuring the intensities and scattering angles of the X-rays that leave the material [156]. This technique is widely used for the analysis of NP phase and crystallinity. However, the resolution and accuracy of XRD can be affected in cases where the samples have highly amorphous characteristics with varied interatomic distances or when the NPs are smaller than several hundreds of atoms [25].

Examples: For the characterization of biogenic Ag NPs, the XRD results of Ag NPs produced by *Trichoderma koningii* [142], *Solanum tuberosum* [143], and *Acanthospermum hispidum* leaf extract [153] displayed characteristic peaks occurring at roughly 2θ = 38 ^o^, 44°, and 64^o^ corresponding to (111), (200), and (220) planes, respectively. These results are in good agreement with the reference to the face-centered cubic structure of crystalline silver. However, the XRD results of Ag NPs produced by *Solanum tuberosum* were not as clear as the other biogenic Ag NPs and had several impurities. The structural characterization of Pd NPs produced by *Garcinia pedunculata* Roxb leaf extract by XRD showed the distinct peaks of Pd, however, three other peaks were also observed at 2θ of 34.22˚, 55.72˚, and 86.38˚, indicating the presence of PdO phases along with Pd NPs [157].

##### Energy-dispersive X-ray spectroscopy (EDX)

This technique is based on the irradiation of the sample with an electron beam. Electrons of the electron beam when incident on the sample surface eject inner shell electrons, the transition of outer shell electrons to fill up the vacancy in the inner shell produces X-rays. Each element produces a characteristic X-ray emission pattern due to its unique atomic structure, and therefore can be used to perform compositional analysis [158]. The shortfall of EDX is that the resulting spectra give only qualitative compositional information (it shows the chemical elements present in the sample without quantification). However, the peak intensities to some extent give an estimate of the relative abundance of an element in a sample [159]. This technique does not require sophisticated additional infrastructures, usually it is a small device that is connected to an existing SEM or TEM. This allows the use of SEM or TEM for the morphological characterization and EDX is used simultaneously for the analysis of chemical composition [160].

Examples: The EDX technique is usually used for the confirmation of the presence of the element in question in biogenic NPs. For instance, EDX was used to confirm the presence of Au in Au NPs produced by *Jasminum auriculatum* leaf extract [161], the presence of Pd in Pd NPs produced by *Pulicaria glutinosa* extract [162], the presence of Te in Te NPs produced by *Penicillium chrysogenum* PTCC 5031 [163], and the presence of Ag in Ag NPs produced by *Trichoderma viride* [164].

##### High-angle annular dark-field imaging (HAADF)

This method is used for the elemental mapping of a sample using a scanning transmission electron microscope (STEM). The images are formed by the collection of incoherently scattering electrons with an annular dark-field detector [165]. This method offers high sensitivity to variations in the atomic number of elements of the sample, and it is used for elemental composition analysis usually when the NPs of interest consist of relatively heavy elements. The contrast of the images is strongly correlated with atomic number and specimen thickness [166].

Examples: The employment of HAADF-STEM in the characterization of biogenic Au–Ag–Cu alloy NPs confirmed the presence of the three elements in the same NP [167]. Similarly, this approach revealed that Ag NPs produced by *Andrographis paniculata* stem extract were coated with an organic polymer [168]. The employment of this approach in the characterization of Cu NPs produced by *Shewanella oneidensis* revealed that Cu NPs remained stable against oxidization under anaerobic conditions, but when they were exposed to air a thin shell of Cu_2_O develop around the NPs [169].

##### X-ray photoelectron spectroscopy (XPS)

This technique is considered the most sensitive approach for the determination of NP exact elemental ratios, chemical state, and exact bonding nature of NP materials [25]. XPS is based on the photoelectric effect that can identify the elements within a material, or covering a material, as well as their chemical state with high precision [170]. XPS can also be used to provide in-depth information on electron transfer, e.g., for Pt NPs supported on CeO_2_, it was found that per ten Pt atoms only one electron is transferred to the support [171].

Examples: The XPS technique can employed for different purposes. For instance, it was used for measuring the purity of Au NPs produced by cumin seed powder [172]. XPS was used for the determination of the oxidation states of Pt NPs produced by *Nigella sativa* seeds and Ag NPs produced by *Rosa canina*. XPS results of Pt NPs showed the presence of three oxidation states for Pt (Pt (0), Pt (II), and Pt (IV)) and two oxidation states for Ag NPs (Ag (0) and Ag (I)). In both cases, the zero-oxidation state was the abundant one, the presence of a small amount of the other oxidation states suggests that some of the NPs were oxidized or had unreduced species [173, 174]. XPS was used for the determination of the exact elemental ratios and the bonding nature of FeS NPs produced by *Shewanella putrefaciens* CN32. For the exact elemental ratios, the researchers compared biogenic and abiotic FeS NPs and found that biogenic FeS NPs had a 2.3:1 Fe:S ratio while the abiotic NPs had a 1.3:1 Fe:S ratio. For the bonding nature, it was determined that the surface of NPs had Fe(II)-S, Fe(III)-S, Fe(II)-O, and Fe(III)-O bonds [175].

#### Fourier-transform infrared spectroscopy (FTIR)

This technique is based on irradiating a material with infrared light, where the absorbed or transmitted radiation is recorded. The resulting spectrum represents a unique fingerprint of samples, where information about the nature of the sample can be obtained such as the bonds involved, polarity, and oxidation state of the sample [176, 177]. This technique is mainly used for the characterization of organic materials such as the surface chemical composition or functionalization of NPs. It is also used for the identification of contaminants when high purity is sought [178].

Examples: For biogenic NPs, FTIR is usually used for the identification of probable functional groups present on the surface of NPs that are responsible for the reduction and stabilization of the NPs. For plant-mediated NP synthesis, for instance for Ag NPs produced by *Camellia sinensis*, the FTIR results indicate the presence of *Camellia sinensis* phytocompounds, such as caffeine and catechin, on the surface of Ag NPs that could be responsible for the reduction of Ag or act as stabilizing agents [147]. For Ag NPs produced by *Solanum tuberosum*, the NPs were found to be capped by amide and amine groups [143]. For CeO_2_ NPs produced by *Eucalyptus globulus*, the polyphenol groups present in *Eucalyptus globulus* extract were found on the surface of NPs suggesting their involvement in the reduction/stabilization process [150]. For microbe-mediated NP synthesis, FTIR results show the presence of protein residues on the surface of NPs confirming the involvement of different proteins in the reduction/stabilization process, such as in Ag NPs produced by *Streptomyces sp.* NH28 [179], in Te NPs produced by *Penicillium chrysogenum* PTCC 5031 [163], and in Se NPs produced by *Azospirillum thiophilum* [180].

##### Zeta potential analysis

Zeta potential measurements are used for the determination of NP surface charge in colloidal solutions. The surface charge of NPs attracts counter-ions that form a thin layer on the surface of the NPs (called Stern layer). This layer travels with the NPs as they diffuse thought the solution. The electric potential at the boundary of this layer is known as NP zeta potential [136]. The instruments used to measure this potential are called zeta potential analyzers [181]. Zeta potential values are indicative for NP stability, where higher absolute value of zeta potential indicate more stable NPs [136].

Examples: The zeta potential is a good indicator for the stability of NPs, where NPs with zeta potentials of more than + 30 mV or less than − 30 mV are considered stable. Zeta potentials have been measured for a wide range of biogenic NPs. The zeta potential for Ag NPs produced by *Ziziphus jujuba* leaf extract of − 26.4 mV [182]. Ag NPs produced by other organisms have different zeta potential values, for example, Ag NPs produced by *Punica granatum* peel extract have a zeta potential of − 40.6 mV indicating their higher stability [183], while Ag NPs produced by *Aspergillus tubingensis* have a zeta potential of + 8.48 indicating their relative instability [184]. The pH of the sample is another important parameter for zeta potential values, the higher pH the lower the zeta potential value [185]. Having different zeta potential values for the same type of NPs depending on the organism used for their synthesis is not unique to silver, Se NPs also show different potential values depending on the organism used for their synthesis [186].

##### Cyclic voltammetry (CV)

CV is an electrochemical technique for measuring the current response of redox-active solutions to a linearly cycled potential sweep between two or more set values. The CV technique involves the use of three electrodes: a working electrode, reference electrode, and counter electrode. These electrodes are introduced to an electrochemical cell filled with an electrolyte solution and where voltage is in excess, the potential of the working electrode is cycled and the resulting current is measured. This technique is used for determining information about the reduction potential of materials, the kinetics of electron transfer reactions, and the thermodynamics of redox processes [187–189].

Examples: The CV technique can be employed for two different purposes in the context of biogenic NP characterization. Firstly, it can be used for measuring the stability of NPs in electrocatalysis. For this purpose, the biogenic NPs are assembled on an electrode of the electrolysis cell and are tested for their electrocatalytic behavior against a redox reaction over different cycles. As an example, Ag NPs produced by *Citrus sinensis* were found to be stable in phenolic compounds redox reactions over multiple cycles [190]. Secondly, CV can be used for monitoring the progress of reduction of metallic NPs or for the determination of the reducing agent involved in the reduction. For example, for Ag NPs produced by Indian propolis, four cyclic voltammograms were recorded, one for a water extract of Indian propolis, another for an ethanol extract of Indian propolis, and two for the constituent flavonoids of Indian propolis (pinocembrin and galangin). The four cyclic voltammograms showed similar behaviors indicating the involvement of these flavonoids in the reduction of Ag and in forming Ag NPs [191].

##### Raman spectroscopy

This technique is based on irradiating a sample with monochromatic light emitted by a laser, in which the interactions between the laser light and molecular vibrations (photons and phonons) are recorded. The technique records the inelastically scattered photons, known as Raman scattering (named after the Indian physician C. V. Raman) [192]. The output of this technique is a unique fingerprint for each sample, which is used to characterize the chemical and intramolecular bonding of the sample. It can also be used to characterize the crystallographic orientation of the sample [193]. Surface-enhanced Raman spectroscopy (SERS) enhances Raman scattering of a sample and provides a more sensitive, specific, and selective technique for identifying molecular structures [194]. Both techniques are also used for the characterization of optical properties, where the recorded photons and phonons are used to understand the plasmonic resonance of NPs [25].

Examples: Raman spectroscopy was used to characterize Fe_3_O_4_ NPs produced by *Pisum sativum* peel, the researchers found that the NPs were Fe_3_O_4_ NPs with face centered cubic phase which was in agreement with their XRD measurements [195]. Other researchers used Raman spectroscopy for studying the trace deposits of carbohydrates on ferrihydrite NPs produced by *Klebsiella oxytoca*, the results showed that the pores of NPs had more deposits of carbohydrates that the surface of the NPs [196]. For Au NPs produced by *Raphidocelis subcapitata* (green algae), several biomolecules were suggested for their involvement in this process. SERS technique was used to study Au NPs surface-associated biomolecules in order to narrow down the list of biomolecules involved in the bioproduction process. The researchers found that several biomolecules such as, glutathione, β-carotene, chlorophyll a, hydroxyquinoline, and NAD were associated with Au NPs surface, thus, ruling out other molecules such as, glutaraldehyde fixing agent, saccharides, FAD, lipids, and DNA from the list [197].

#### Characterization of optical, electronic, and electrical properties

In addition to Raman spectroscopy and SERS, also other techniques can be employed to study and characterize the optical properties of NPs. These techniques give information about the absorption, reflectance, fluorescence, luminescence, electronic state, bandgap, photoactivity, and electrical conductance properties of NPs.

##### Ultraviolet–visible spectroscopy (UV–vis) and photoluminescence spectroscopy (PL)

In absorption spectroscopy such as UV–vis, the transition of electrons from the ground state to an excited state is measured, while in photoluminescence spectroscopy, the transition of electrons from the excited state to the ground state is measured [198]. UV–vis spectroscopy uses visible and UV light to measure the absorption or reflectance of a sample. In photoluminescence spectroscopy, usually UV light is used to excite the electron and then measure the luminescence or fluorescence properties of a sample [199].

Examples: UV–vis spectroscopy is a simple and common technique that is used for the characterization of the optical properties of NPs. For instance, for the characterization of the optical properties of Ag NPs produced by *Trichoderma viride*, the UV–vis spectrum showed that a Ag surface plasmon band occurs at 405 nm, which is a characteristic band for Ag NPs. The intensity of this band over the reaction time increased as a result of increasing Ag NP concentration in the solution. In the same study, the photoluminescence properties of these NPs were recorded, with an emission in the range between 320–520 nm, which falls in the blue-orange region [164]. For biogenic Cu NPs, the common absorption peaks are located between 530–590 nm. The difference in NP size and the bio-active molecules used for the reduction process are believed to be the reasons behind the differences in the absorption peaks [200]. For instance, 15 nm spherical Cu NPs produced by *Calotropis procera* have an absorption peak at 570 nm [201], while 76 nm spherical Cu NPs produced by *Duranta erecta* have an absorption peak at 588 nm [202]. The same applies to photoluminescence effects, where 27 nm spherical Cu NPs produced by *Tilia* extract emit light of 563 nm (dark brown) [203], while 19 nm spherical Cu NPs emit light of 430 nm (green) [204].

##### UV–vis diffuse reflectance spectroscopy (DRS)

This technique uses UV and visible light to measure the diffuse reflectance of a material (the reflection of light in many angles, as opposed to specular reflection). The resulting diffuse reflectance spectra are used to determine the electronic state of a sample, which is then used to calculate the bandgap [25]. Bandgap determination is crucial for determining conductance and photocatalytic properties especially for semiconductor NPs [205].

Examples: The DRS technique was used to calculate the bandgap for a wide range of biogenic NPs. For instance, TiO_2_ NPs produced by *Andrographis paniculata* exhibit an optical energy bandgap of 3.27 eV [206]. Interestingly, biogenic ZnO NPs produced by different organism show different bandgaps, for example, ZnO NPs produced by *Pseudomonas putida* have a bandgap of 4 eV [207], while ZnO NPs produced by *Calotropis procera* leaf extract have a bandgap of 3.1 eV [208].

##### Spectroscopic ellipsometry

This technique is based on irradiating a sample with polarized light to measures changes in polarization. It is widely used to calculate the optical constants of a material (refractive index and extinction coefficient) [209]. This technique is also used to characterize the electrical conductivity and dielectric properties of materials [210].

Examples: Spectroscopic ellipsometry is not a common technique for the characterization of biogenic NPs. For chemically produced NPs, the optical properties for different-sized Au NPs partially embedded in glass substrate were measured by spectroscopic ellipsometry. In this example, a clear transition from LSPR to SPR mode was found as the thickness increases. Moreover, the partially-embedded Au NPs had much higher refractive index sensitivity compared to Au NPs fully immobilized in a glass substrate [211]. Spectroscopic ellipsometry was also used to measure the changes in the optical constants of a layer of 5 nm ZnO NPs induced by UV illumination. In this case, it was found that the UV illumination of ZnO NPs in inert atmospheres resulted in a clear blue shift in the absorption (Moss-Burstein shift). The UV illumination of ZnO NPs results in the desorption of O_2_ from the NPs surface leading to the population of the lowest levels in conduction band with mobile electrons. This phenomenon is reversible, in which the exposure to O_2_ from air results in the scavenging of these mobile electrons [212].

#### Characterization of magnetic properties

The magnetic properties of NPs are of high importance, as they potentially give NPs great advantages in catalysis, electronics, and medical applications. Several techniques were developed for the detection and quantification of small magnetic moments in NPs.

##### Magnetic force microscopy (MFM)

**Fig. 7:**
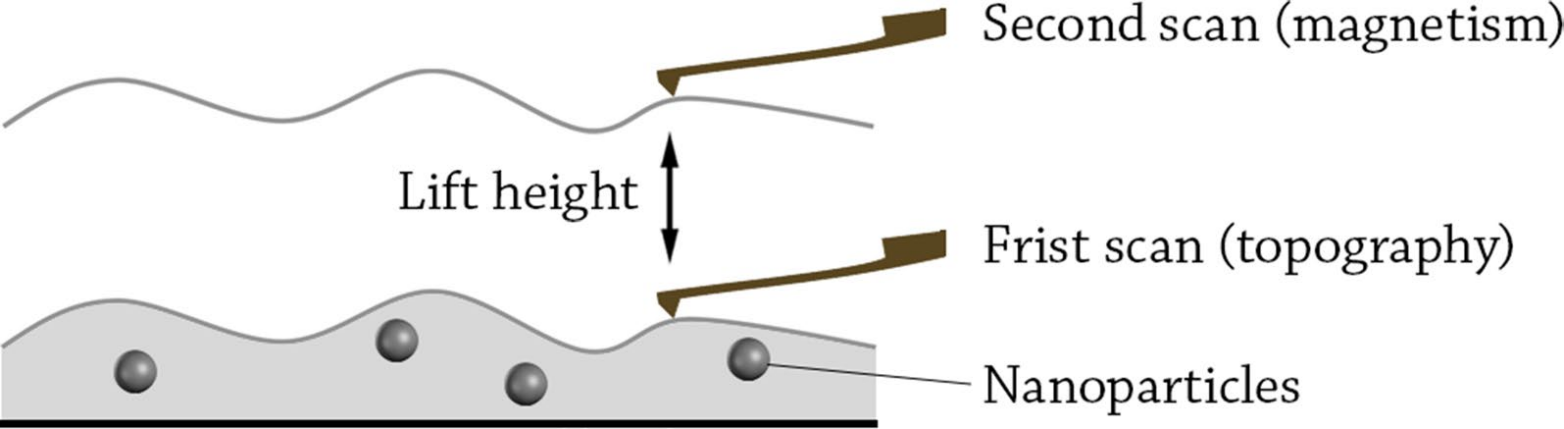
Magnetic force microscopy lift height method. The first scan is done very close to the surface to obtain the topography of the sample. Then, the tip is lifted and a second scan is performed following the topography outline obtained in the first scan

This technique is a variety of atomic force microscopy (AFM), in which a magnetic tip is used to scan the sample. The magnetic tip is approached very close to the sample, where the magnetic interactions between the tip and the sample are recorded [213]. At closer distances to the sample (0–20 nm), other forces such as van der Waals forces also interact with the tip. Therefore, MFM measurements are often operated with two-pass scanning method (also called lift height method) [214] (Fig. 7). In this method, the tip is firstly used to measure the topography of the sample including the molecular forces as van der Waals. Afterwards, the tip is lifted and a second scan is operated following the same topography outline. In the second scan, the short-ranged van der Waals forces disappear and the long-range magnetic forces are almost exclusively recorded. In an experimental study, researchers found that 22 nm was the optimal scanning height for the second scan, at which van der Waals forces are very weak while the distance is still small enough to measure the magnetic interactions for Pd-Fe bimetallic NPs [215].

Examples: MFM was heavily used for the characterization of magnetite NPs produced by magnetotactic bacteria. For instance, the size and orientation of the magnetic moment of magnetite NPs produced by *Magnetospirillum gryphiswaldense* strain MSR-1 were studied by MFM [216], in which the size of the magnetic moment was found to be 1.61 × 10^−17^ Am^2^. In a different study, MFM was used to characterize the magnetic properties and to estimate the size of the magnetic kernel of the magnetosomes produced by the same strain, and it was determined that the NPs behaved like single mono-domain nanomagnets [217]. The magnetic properties of NPs made from materials such as Pd that only exhibit significant magnetism on the nanoscale can also be studied by MFM, however, the magnetic moment of these NPs is much lower than for ferromagnetic NPs. The magnetic decoration of Pd NP samples with Fe_2_O_3_ NPs strongly enhances the weak magnetic signal of Pd NPs up to 15 times [218]. This approach could make the MFM technique useful for the characterization of weak magnetic NPs.

##### Vibrating-sample magnetometry (VSM)

This technique measures the magnetic properties of materials based on Faraday’s law of induction. In VSM, the sample is placed in a constant magnetic field in a special holder that vibrates vertically. As the holder starts vibrating, the magnetic moment of the sample creates a magnetic field that changes as function of time. The alternating magnetic field created in the sample induces an electric current that is recorded and used to calculate the magnetic properties of the sample [219, 220].

Examples: For the characterization of Fe_2_O_3_ NPs produced by *Tridax* leaf extract, VSM studies revealed that the NPs had a saturation magnetization of 7.78 emu/g, a remnant magnetization of 0.054 emu/g, and a coercivity of − 1.6 G [221]. In other studies, VSM was used to compare the magnetic properties of iron oxide NPs produced *Moringa oleifera* with the magnetic properties of the same NPs but coated with chitosan. The researchers found that saturation magnetisation, remnant magnetization, and coercivity have lower values when the NPs are coated with chitosan [222].

##### Superconducting quantum interference device (SQUID) magnetometry

This technique measures the magnetic properties of materials based on the Josephson effect. Niobium (Nb) or other metal alloys are used in the device which needs to be operated at temperatures very close to the absolute zero to main superconductivity, where liquid helium is used to maintain the cold environment [223]. However, other kinds of SQUID also exist where high-temperature superconductors are used [224]. After reaching superconducting environments, the Josephson junctions contained in the device help to create a supercurrent, which is recorded and used to calculate the magnetic properties of the sample [225].

Examples: For the characterization of iron oxide NPs produced by *Cnidium monnieri* seed extract, SQUID magnetometry revealed that the NPs had a saturation magnetization of 54.60 emu/g, a remnant magnetization of 1.15 emu/g, a coercivity of 11 Oe, and a magnetic susceptibility of + 1.69 × 10^–3^ emu/ cm^3^⋅Oe at room temperatures, indicating the superparamagnetic behaviour of these NPs [226]. SQUID magnetometry was also used for the characterization of the magnetic properties of zinc incorporated magnetite NPs produced by *Geobacter sulfurreducens*, showing that the loading of only 5% zinc results in the enhancement of saturation magnetization of the NPs by more than 50% [227].

##### Electron spin resonance spectroscopy (ESR)

This technique measures the magnetic properties of materials by characterizing and quantifying the unpaired electrons in the sample. Electrons are charged particles that spin around their axis, which can align in two different orientations (+ ½ and − ½) when the sample is placed in strong magnetic field. These two alignments have different energies due to the Zeeman effect. Since unpaired electrons can change their spins by absorbing or emitting photons, in ESR the sample is irradiated with microwave pulses to excite electron spins until a resonance state is reached [228]. This technique is also referred to as electron paramagnetic resonance spectroscopy (EPR). It can be used to measure the ferromagnetic and antiferromagnetic properties of NPs [229, 230].

Examples: ESR was used to characterize the magnetic properties of iron oxide NPs produced by *Ficus carica*. The trees naturally produce iron oxide NPs as a defence mechanism when are they are subjected to stress. The researchers found that the magnetic properties of iron oxide NPs produced by the same tree but grown in different environmental conditions have different magnetic properties. In addition, a magnetic anisotropy of the signal was visible as the magnetic properties of these NPs varied strongly at different temperatures [231]. ESR was also used to characterize the magnetic properties of Se nanomaterials produced by anaerobic granular sludge. The ESR results revealed the presence of Fe(III) atoms incorporated in the Se nanomaterial, which enhanced their overall magnetic properties, giving it ferromagnetic behaviour [232].

#### Characterization of thermal properties

Several techniques can be used for the characterization of the thermal properties of NPs, such as melting points, crystallization and structural-phase transition points, heat capacity, thermal conductivity, and thermal and oxidative stability.

##### Differential scanning calorimetry (DSC)

In this technique the analyte and a well-defined reference sample are put at the same temperature, then, the amount of heat required to increase the temperature of the sample and the reference in measured as a function of temperature. This technique is widely used to measure melting points [233], crystallization points, structural-phase transition points [234], latent heat capacity [235], heat of fusion [236], and oxidative stability [237].

Examples: For the characterization of Ag NPs produced by *Rhodomyrtus tomentosa* leaf extract, DSC showed three exothermic peaks at 44, 159, 243, and an endothermic peak at 441 °C. The first peak (at 44 °C) indicates that at this temperature the NPs face a gradual loss of water from their surface. The second peak (at 159 °C) shows that the thermal decomposition of the sample happens at this temperature. The last temperature (441 °C) indicates the melting temperature for those NPs [238]. For Ag NPs produced by *Parthenium hysterophorus* leaf extract, DSC showed that their melting temperature was at 750 °C. The researchers also found that these NPs had completely thermally decomposed and crystallized simultaneously [239].

##### Differential thermal analysis (DTA)

This technique is based on heating or cooling a sample and an inert reference under identical conditions, where any temperature difference between the sample and the reference is recorded. This technique is primarily used for the study of phase diagrams and transition temperatures [240]. However, it is also used to measure the melting points, thermal, and oxidative stability [241, 242].

##### Thermogravimetric analysis (TGA)

This technique measures the change in the mass of a sample as a function of temperature and/or time in a controlled atmosphere [243]. This technique is mainly used to study the thermal stability of materials [244], in addition, it is also used to measure structural-phase transition points [245], thermal activation energies [246], and oxidative stability [247]. The resulting thermogram is unique for each compound and therefore can also be used for the determination of material composition [248]. TGA and DTA are usually combined in the same thermal analyzing instrument, called thermogravimetry/differential thermal analysis (TG/DTA) [244].

Examples: TG/DTA is a common technique for the characterization of thermal properties of biogenic NPs. For instance, the thermal properties of Ag NPs produced by *Daphne mucronate* leaf extract were studied in the range between 0–1000 °C where the sample was heated at a rate of 10 °C/min. The researchers found that between 400–500 °C the NPs faced a dominant weight loss, while the weight loss below 400 °C and above 500 °C was negligible. The DTA curve showed an intense exothermic peak in the range between 400–500 °C, this indicates that the crystallization of NPs happens in this temperature interval. Some minor weight loss events were seen below 400 °C, this may be caused by the evaporation of water or the degradation of the organic components [249]. In another study, the thermal properties of Ag NPs produced by two different plants (*Stereospermum binhchauensis* and *Jasminum subtriplinerve*) were compared. The researchers found that the major weight loss happens between 220–430 °C, which is attributed to the decomposition of biomolecules from the NP surface [250]. This shows that Ag NPs produced by these plants have much higher content of biomolecules on their surface than Ag NPs produced by *Daphne mucronate.* TG/DTA showed that *Stereospermum binhchauensis* Ag NPs crystallize at 315 °C and *Jasminum subtriplinerve* Ag NPs at 345 °C, around 100 °C less than *Daphne mucronate* Ag NPs [250].

##### Transient hot wire method (THW)

This method is used for the determination of thermal conductivity based on increasing the temperature of a material by a thin hot wire as a function of time, where the heating wire is located directly in the test sample. The advantage of this method over other thermal conductivity measurement methods is the very short measuring time, this gives high accuracy of thermal conductivity due to the negligible values of convection in such short times [251]. In this method, the NPs are added to a solution (usually water or ethylene glycol) forming a colloidal dispersion called a nanofluid. Then, the thermal conductivity of the nanofluid is measured and compared to the thermal conductivity of the base fluid, giving a thermal conductivity ratio which is used to evaluate the thermal conductivity of different NPs.

Examples: The thermal conductivity ratios of three different concentrations (0.12, 0.18, and 0.24%) of biogenic SnO_2_ NPs produced by *Punica granatum* seed extract were measured in ethylene glycol at 303 K. The researchers found a linear relationship between NPs concentration and the thermal conductivity. The thermal conductivity enhancement of nanofluid to base fluid was between 6 and 24% [252]. In another study, the thermal conductivity of Fe_2_O_3_ NPs produced by *Psidium guajava* leaf extract was measured in water and in ethylene glycol. The researchers found that the thermal conductivity enhancement in ethylene glycol was better than in water, the thermal conductivity enhancement for 0.025% Fe_2_O_3_ NPs in water was 30% while in ethylene glycol was 34%. Moreover, the linear relationship between NPs concentration and thermal conductivity ratio was found for Fe_2_O_3_ NPs in both water and ethylene glycol [253].

#### Characterization of mechanical properties

Several methods can be used for the characterization of mechanical properties of NPs, such as tensile and compressive strengths, elasticity, viscoelasticity, hardness, and stiffness.

##### Tensometery

The machine used for this method is called a universal testing machine (UTM) or a tensometer. It is used to measure the elasticity (elastic modulus), tensile and compressive strengths (Young’s modulus) of materials. In this machine, the sample is placed between grips and an extensometer, where changes in gauge length are recorded as a function of load [254]. However, other mechanical changes in addition to the change in gauge length are also recorded in this machine, such as the elasticity.

Examples: The mechanical properties of different biogenic NP-containing composites can be measured by this machine. For example, the mechanical properties of orthodontic elastic ligatures containing Ag NPs produced by *Heterotheca inuloides* were studied by comparing the maximum strength, tension, and displacement of the composite with and without the biogenic NPs. The researchers found that maximum strength, tension, and displacement have improved after the addition of Ag NPs [255]. Interestingly, the addition of biogenic Ag NPs produced by *Diospyros lotus* fruit extract to starch and polyvinyl alcohol hydrogel membranes resulted in an adverse effect. The tensile strength and modulus of the hydrogel membranes containing 50 and 100 ppm Ag NPs were much lower than of the neat hydrogel membrane. The researchers attributed this adverse effect to the possibility that the addition of Ag NPs could have resulted in blocking the crosslinking between starch and polyvinyl alcohol, or to the possibility of the formation of breakage points in the polymer matrix due to NPs agglomeration [256].

##### Instrumented indentation testing

This method is used to characterize the hardness features of materials by using a well-defined hard indenter tip typically made of diamond. The indenter tip is used to make an indentation in the sample by placing incremental loads on the tip, after which the area of indentation in the sample is measured and used to calculate the hardness features [257]. Light microscopy, SEM, or ATM technique are usually used to visualize the indentation in the sample. The method is also called micro- or nano-indentation testing.

Examples: This method was used to characterize the mechanical properties of calcite NPs produced by *Ophiocoma wendtii* brittlestar. The arm plates of this brittlestar are covered by hundreds of nanoscale calcite lenses that focus light onto photoreceptor nerve bundles positioned beneath the brittlestar. The researchers used the nanoindentation method to compare Young’s modulus, hardness and fracture toughness of biogenic calcite with geocalcite. The results showed that the biogenic calcite lenses have higher hardness and fracture toughness compared to geocalcite (more than twofold) [258]. Bamboo is well known for its high silica content in comparison to other wood species. It produces SiO_2_ NPs and deposits it in its epidermis in the form of silica cells. The mechanical properties of silica cells compared to other types of cells of Moso bamboo (*Phyllostachys pubescens*) were studied by instrumented indentation testing. The researchers found that the cell wall of silica cells display higher hardness and elastic recovery compared to fibre and epidermal cells, which is attributed to the presence of biogenic SiO_2_ NPs in the silica cells [259].

##### Dynamic mechanical analysis (DMA)

This method is used to study the mechanical properties of materials by measuring the strain of a material after applying a stress. This method helps to obtain three different values: storage modulus, loss modulus, and loss tangent. These values are important to give an overview about the stiffness and viscoelasticity behavior of materials [260].

Examples: The DMA method was used to characterize the mechanical properties of polymethyl methacrylate denture base polymer filled with Ag NPs produced by *Boesenbergia rotunda*. In this study frequency sweep test was used to determine the viscoelastic behavior of this nanocomposite where the temperature was constant at 37 °C and the frequency was increasing from 0.5 to 100 Hz in tension mode. The researchers found a frequency dependence for storage modulus, loss modulus, and loss tangent for the nanocomposite with various Ag NPs loading concentrations. The frequency dependence of storage modulus, loss modulus, and loss tangent indicates the viscoelastic response of this polymer. However, the results showed that the storage modulus for the nanocomposite is much higher than the loss modulus over the range of frequencies, indicating the elastic dominance of the nanocomposite. Moreover, the researchers found that storage and loss moduli increase with increasing Ag NPs loading concentrations, which is due to the interaction between polymethyl methacrylate and Ag NPs [261].

In a different study, DMA was used to determine the thermomechanical properties of pol(S-co-BuA) polymer filled with cellulose nanocrystals produced by *Posidonia oceanica*. In this case, the behaviour of storge modulus and loss tangent were studied as a function of temperature for different cellulose nanocrystals loading concentrations. The results showed that the unloaded polymer behaves like an amorphous polymer, the storage modulus remains constant until the temperature reaches 25 °C then it starts to sharply decrease due to glass–rubber transition. A relaxation process was also evident for the unloader polymer, where the loss tangent reaches its maximum at 35 °C then it starts to fall. The addition of cellulose nanocrystals to the polymer positively enhanced both effects. The dramatic drop of storage modulus at 25 °C was less for the nanocomposite, where the drop for the polymer loaded with 15% cellulose nanocrystals was almost cancelled. Similar positive enhancement was found for loss tangent. These enhancements could be attributed to the mechanical coupling effect, in which the NPs connect and form a stiff continuous network linked through hydrogen bonding [262].

## Applications of NPs

NPs, due to their above-mentioned unique or enhanced physicochemical properties, are used in a wide range of applications in different fields. In addition, several potential applications are in research and development. Here we present some examples of these applications.

### Applications in medicine and pharma

Metallic and semiconductor NPs have huge potential for cancer diagnosis and therapy based on their enhanced light scattering and absorption properties due to LSPR effect. For instance, Au NPs efficiently absorb light and convert it into localized heat, which can be exploited for selective photothermal therapy of cancer (cancer cell death by heat generated in tumor tissue) [263, 264]. In addition, the unique optical properties of Au NPs make them a great candidate for the photodynamic therapy of cancer (the use of a drug that is activated by light to kill cancer cells) [265]. Gd based NPs have also shown great abilities in tumor growth inhibition [266], metastasis inhibition [267], and tumor-specific magnetic resonance contrast enhancement [268]. Targeted drug delivery is also an important potential application of NPs. ZnO and Fe_3_O_4_ NPs were efficiently used for targeted drug delivery and selective destruction of tumor cells [269–271].

Moreover, NPs have been successfully used in different medical applications such as cellular imaging [272], or in biosensors for DNA, carbohydrates, proteins, and heavy metal ions [273, 274], determination of blood glucose levels [275], and for medical diagnostics to detect bacteria [276] and viruses [277]. For instance, Au NPs were conjugated with SARS-CoV-2 antigens to rapidly detect the presence of SARS-CoV-2 IgM/IgA antibodies in blood samples within 10–15 min [278], At the same time, due to their antimicrobial and antibacterial activities, NPs such as TiO_2_, ZnO, CuO, and BiVO_4_ are being increasing used in various medical products such as catheters [279, 280].

### Applications in electronics

NPs, due to their novel electronic and optical properties, have a wide range of potential applications in imaging techniques and electronics. For instance, Gd-based NPs can improve the imaging quality and the contrast agent administration dose of magnetic resonance imaging (MRI). The use of Gd_2_O_3_ NPs as a contrasting agent was found to be more efficient than the commonly used agent (Gd-DOTA) at the same concentration [281]. At the same time, GdPO_4_ NPs were successfully used for tumor detection using MRI in 1/10 of the dose typically used with Gd-DTPA agent [282]. Interestingly, NPs also offer the ability to image and track a single molecule, which can reveal important information about cellular processes such as membrane protein organization and interaction with other proteins. For example, Eu^3+^-doped oxide NPs were used to track a single toxin receptor with a localization precision of 30 nm [283].

Regarding applications in batteries, an important component in lithium-ion batteries is the separators. Their main function is to prevent the physical contact of anode and cathode, and to provide channels for the transport of ions. The commonly used commercial material in battery separators, a polyolefin microporous membrane, suffers from poor electrolyte uptake and poor thermal stability [284]. Due to the aerogel structure of some NPs (such as ZnO NPs), they are an ideal choice for separator plates in batteries [284]. This makes the batteries store a significantly higher amount of energy compared to traditional batteries. For lithium-air batteries, using Pt-Au bimetallic NPs strongly enhances oxygen reduction and oxygen evolution reactions [285]. Moreover, batteries made of nanocrystalline Ni and metal hydrides last longer and require less charging [23]. In addition to battery applications, several NPs such as CdS and ZnSe are also used in light-emitting diodes (LED) of modern displays to get higher brightness and bigger screens [23, 286]. Other NPs such as CdTe NPs are also used in liquid crystal displays (LCDs) [287]. The addition of a NP layer to LED and LCD enables them to generate more light using the same amount of energy and enhances their lifetime.

### Applications in agriculture

NPs have potential to benefit the agriculture field by providing new solutions to current agricultural and environmental problems [288]. NPs are mainly used in two forms in agriculture, as nanofertilizers and nanopesticides. Chemical fertilizers have poor efficiency due to leaching and volatilization. In these cases, the farmers usually react by using excessive amounts of fertilizers, which increases crops productivity but has an environmental cost [288]. In contrast, nanofertilizers are compounds that are applied in smaller amounts than regular chemical fertilizers but yet have better efficiencies [289]. The difference in efficiency comes from the fact that they are able to release the nutrients just when and where they are required by the plants. In that way, they limit the conversion of excess amounts of fertilizer to gaseous forms or from leaking into the ground water [290]. Several NPs have been employed in the development of fertilizers, including SiO_2_, ZnO, CuO, Fe, and Mg NPs [291–293]. These nanofertilizers provide the plants with increased nitrogen fixation, improved seed germination, amelioration to drought stress, increased seed weight, and increased photosynthesis ability [291–293]. The large surface area and small size of these NPs are the main reasons for the better efficiencies of nanofertilizers over conventional fertilizers [294].

Several NPs have proven antimicrobial, insecticidal, and nematicidal activities, which makes them a promising alternative to chemical pesticides and a potentially cheaper alternative to biopesticides [294]. For instance, the photocatalytic activity of TiO_2_ NPs gives them a potent antimicrobial activity against *Xanthomonas perforans*, the causing agent of tomato spot disease [295]. CuO NPs show insecticidal activity against *Spodoptera littoralis*, known as African cotton leafworm [296]. Ag NPs show nematicidal activity against *Meloidogyne spp.*, root-knot nematodes [297].

### Applications in the food industry

NPs, despite toxological concerns, have impactful applications in several food industry-related process such as food production, preservation, and packaging. TiO_2_ NPs are a major promising player in this industry. Their photocatalytic antimicrobial activity makes them an interesting material for food packaging [298]. In addition, they are also used in sensors to detect volatile organic compounds [299]. Ag NPs are also promising in food packaging due to their antimicrobial activity. They play an important role in reducing the risk of pathogens and extending food shelf-life [294]. The efficiency of doping Ag and ZnO NPs to degradable and non-degradable packaging materials for meat, bread, fruit, and dairy products was tested against several yeast, molds, aerobic, and anaerobic bacteria [300]. For instance, polyvinyl chloride doped with Ag NPs was evaluated for packing minced meet at refrigerator temperature (4 °C); the results showed that Ag NPs significantly helped to slow down bacterial growth, increasing the shelf-life of minced meet from 2 to 7 days [301].

## Effects of NPs on biological systems

Although the use of NPs is exponentially growing, their possible toxicological and hazardous impacts to human health and environment cannot be ignored. NPs may get released to the environment during production stages, usage, recycling, or disposal. These NPs may persist in air, soil, water, or biological systems [302]. NPs can enter the human or animal body though the skin, orally, or via the respiratory tract, and afterwards move to other parts of the body. The exposure to NPs was found to activate proinflammatory cytokines and chemokines with recruitment of inflammatory cells, which impacts the immune system homeostasis and can lead to autoimmune, allergic, or neoplastic diseases [302]. Moreover, the exposure to ultrafine particles can cause pulmonary, cardiac, and central nervous system diseases [303–305]. Similarly, NPs can enter plants cells and cause harmful effects [306]. For instance, the exposure of ZnO and Al NPs was found to cause root growth inhibition in plants [307, 308].

## Conclusion

Nanoscience and nanotechnology are inherently transdisciplinary fields of science. With new bio-based approaches, there is a need for biologists to understand not only the basic principles of nanoscience, but also the technologies and methods traditionally employed to characterize nanomaterials. We hope that this review can help to inspire new collaborations across different scientific disciplines, by helping biologists to identify the best technologies—and partners—to characterize their nanomaterials. At the same time, we recommend to take potential biological risks of these new materials into careful consideration already during the planning phase of such experiments.

## Data Availability

Not applicable.

## Abbreviations

AFM: Atomic force microscopy
BET: Brunauer–Emmett–Teller
BJH: Barrett–Joyner–Halenda
CV: Cyclic voltammetry
DLS: Dynamic light scattering
DLVO: Derjaguin–Landau–Verwey–Overbeek
DMA: Dynamic mechanical analysis
DMT: Derjaguin–Muller–Toporov
DRS: UV–vis diffuse reflectance spectroscopy
DSC: Differential scanning calorimetry
DTA: Differential thermal analysis
EDX: Energy-dispersive X-ray spectroscopy
EM: Electron microscopy
EPR: Electron paramagnetic resonance spectroscopy
ESR: Electron spin resonance spectroscopy
FTIR: Fourier-transform infrared spectroscopy
HAADF: High-angle annular dark-field imaging
ISO: International Organization for Standardization
JKR: Johnson–Kendall–Roberts
LCD: Liquid crystal display
LED: Light-emitting diode
LSPR: Localized surface plasmon resonance
MFM: Magnetic force microscopy
MRI: Magnetic resonance imaging
NPs: Nanoparticles
NTA: Nanoparticle tracking analysis
PL: Photoluminescence spectroscopy
r_c_: Critical radius
r_sp_: Threshold radius for superparamagnetism
SAED: Selected area electron diffraction
SEM: Scanning electron microscopy
SERS: Surface-enhanced Raman spectroscopy
SPR: Surface plasmon resonance
SQUID: Superconducting quantum interference device
STEM: Scanning transmission electron microscopy
STM: Scanning tunneling microscopy
TEM: Transmission electron microscopy
TG/DTA: Thermogravimetry/differential thermal analysis
TGA: Thermogravimetric analysis
THW: Transient hot wire
UTM: Universal testing machine
UV: Ultraviolet
UV–vis: Ultraviolet–visible spectroscopy
VSM: Vibrating-sample magnetometry
XPS: X-ray photoelectron spectroscopy
XRD: X-ray diffraction analysis

## Footnotes

1
https://www.etymonline.com/word/nano.

2 [SOURCE: ISO/TS 80,004‑2:2015, 4.4].
